# Association between depressive symptoms and atherosclerotic cardiovascular disease: a Swedish population-based cohort study

**DOI:** 10.1186/s12872-026-05629-8

**Published:** 2026-05-13

**Authors:** Christian Rausch, Saadbin Zafar Mahmood, Yajun Liang, Jette Möller, Aysha Almas

**Affiliations:** 1 Department of Global Public Health, Karolinska Institutet, Stockholm, Sweden; 2 Acute and Reparative Medicine Theme, Karolinska University Hospital, Stockholm, Sweden; 3 Department of Medicine, Aga Khan University, Karachi, Pakistan; 4 Department of Global Public, Health Epidemiology and Public Health Intervention Research (EPHIR), Karolinska Institutet, Solnavägen 1E, 6:th floor, 113 65 Stockholm, Sweden

**Keywords:** Depression, Mental health, Depressive symptoms, Anxious distress, Atherosclerotic cardiovascular disease

## Abstract

**Background:**

Atherosclerotic cardiovascular disease (ASCVD) remains a leading cause of morbidity and mortality globally. While traditional risk factors such as hypertension, smoking, and dyslipidemia are well established, the role of depressive symptoms in ASCVD development, particularly in premature cases, is less understood. This study aims to determine the association between depressive symptoms, including severity and anxious distress as specifiers, with ASCVD and premature ASCVD in a Swedish population-based cohort.

**Methods:**

We utilized data from the PART study (acronym in Swedish for: Psykisk hälsa, Arbete och RelaTioner: Mental Health, Work and Relationships), a longitudinal cohort study which include a total of 11,175 adults in Stockholm, Sweden. Depressive symptoms were assessed using the Major Depression Inventory across three waves from 1998 to 2010 Incident ASCVD cases were identified through the National Patient Register (NPR) until 2021. Premature ASCVD was defined as occurring before age 55 in men and 65 in women. Cox regression models were used to estimate hazard ratios (HRs) and 95% confidence intervals (95% CIs), adjusting for sociodemographic factors, lifestyle behaviors, and cardiometabolic conditions.

**Results:**

Participants with depressive symptoms had a 42% increased risk of ASCVD (HR 1.42, 95% CI 1.19–1.68), independent of traditional cardiovascular risk factors. When stratifying by ASCVD type, depressive symptoms were associated with ischemic heart disease (HR 1.39, CI 1.06–1.83) and stroke (HR 1.53, CI 1.15–2.05). The risk varied by severity, with mild depressive symptoms associated with ischemic heart disease (HR 1.62, CI 1.11–2.37) and moderate (HR 1.70, 95% CI 1.04–2.78) and severe depressive symptoms (HR 1.61, 95% CI 1.04–2.49) were associated with stroke. Furthermore, participants with combined depressive symptoms and anxious distress had an increased risk of stroke (HR 1.50, CI 1.09–2.07), whereas participants with depressive symptoms without anxious distress had an increased risk of ischemic heart disease (HR 1.88, CI 1.25–2.82). The risk of premature ASCVD was increased among individuals with depressive symptoms (HR = 1.52, 95% CI 1.12–2.05). When examined by level of severity, only among subjects with mild depressive symptoms, a statistically significant association with premature ASCVD (HR = 1.61, 95% CI 1.05–2.48) was found.

**Conclusions:**

This study provides evidence linking depressive symptoms to ASCVD and premature ASCVD in a Swedish population. The findings underscore the need to consider the effects of depressive symptoms as well as potential prevention strategies for depressive symptoms in regard to cardiovascular diseases. Future research should explore whether early identification and treatment of depressive symptoms can mitigate ASCVD risk and improve long-term cardiovascular outcomes.

**Supplementary Information:**

The online version contains supplementary material available at 10.1186/s12872-026-05629-8.

## Background

Atherosclerotic Cardiovascular Disease (ASCVD) stands as a predominant global public health issue, leading in both mortality rates and economic burden on healthcare systems, while significantly impacting disability-adjusted life years (DALYs) [1, 2]. Between 1980 and 2021, Sweden experienced a substantial decline in overall ASCVD morbidity and mortality, reflecting improvements in prevention, treatment, and public health policy [3]. Despite this long-term downward trend, ASCVD remains one of the leading causes of death nationally. In 2021, the highest regional mortality rate for ischemic heart disease was 129.6 per 100,000 population [3]. Importantly, while overall mortality has decreased, no meaningful decline has been reported in premature ASCVD, a cohort for which ischemic heart disease accounts for 12.4% of deaths [4–7].

Studies have identified distinct risk factors for premature compared to later in life occurring ASCVD (non-premature ASCVD), with smoking, family history, and dyslipidemia being more common in premature cases, and hypertension, obesity, sedentary lifestyle, and diabetes mellitus more common in ASCVD occurring later in life (non-premature ASCVD) [3–7]. The multifactorial nature of premature ASCVD, particularly the role of genetic predisposition and family history in younger patients, underscores the complexity of this condition. Various studies have been conducted to determine the significance of various ASCVD risk factors in the Nordic region using population registries and longitudinal databases. In line with the aforementioned risk factors, literature also concludes that patients with a low socioeconomic position, severe mental illness, or having a migrant background receive inadequate cardiovascular care and acute care leading to impaired cardiovascular health [8, 9].

Recent studies have identified depression as a significant, yet often overlooked, factor contributing to ASCVD across genders and living environments [10, 11]. This association is believed to stem from depression’s multifaceted impact on cardiovascular health, including potential changes in cardiac function and rhythm, increased coagulability and platelet aggregation, dysregulation of the hypothalamic-pituitary-adrenocorticalaxis, heightened inflammation, elevated autonomic tone, and endothelial dysfunction [10, 11]. Lifestyle choices in particular smoking and physical inactivity exacerbates the risk by negatively affecting the previously mentioned physiological factors like obesity and hypertension, underscoring the need for lifestyle modifications to prevent cardiovascular complications [12–17].

Previous studies has shown an association between depression and ASCVD [10, 12–14]. Longitudinal cohort studies from Sweden show that persons affected by depression, particularly ones with a more severe level of depression are at increased risk of ASCVD [12, 13]. We have previously reported, from the PART study (in Swedish short for: Psykisk hälsa, Arbete och RelaTioner) that depressive symptoms were associated with future risk of ASCVD, particularly among individuals with more severe depressive symptoms. However, earlier analyses did not examine premature ASCVD. Given the recognized prognostic importance of anxious distress in depressive disorders and the public health significance of premature ASCVD, the present study extends previous findings by incorporating longer follow-up, a larger sample, and detailed assessment of both severity of depressive symptoms and anxious distress [15].

This study aims to determine, the risk of ASCVD and premature ASCVD associated with depressive symptoms in a Swedish cohort, and further to determine if the risk differs across severity of depressive symptoms and anxious distress.

## Methods

### Population and sample

#### Study design

This study is based on data from the PART study (In Swedish short for: Psykisk hälsa, Arbete och RelaTioner) which is a population-based longitudinal cohort study in Stockholm County designed to investigate mental health, work, and social relationships in the general adult population. Data were collected in three waves wave 1 (W1, 1998–2000), wave 2 (W2, 2001–2003), and wave 3 (W3, 2010). All participants with available depression status in W1, W2, or W3 were followed for ASCVD until 2021. Participants with a history of ischemic heart disease or stroke at baseline (*n* = 278) were excluded from the analyses.

In W1,19,457 participants aged 20–64 years were randomly selected and invited to complete postal questionnaires covering sociodemographic characteristics, lifestyle factors, and symptoms of mental and physical ill-health. All participants who responded in W1 received an almost identical questionnaire in W2 and those who responded in W2 received a questionnaire in W3. The date of questionnaire submission was used as the inclusion date. From 19,457 individuals who were invited in W1, 10,411 (53%) agreed to participate. Of them, 8622 (84%) participated in W2 and 5228 (60%) in W3. Non-response analyses has shown that participation was higher among females, higher age, higher income and education, as well as among individuals without a previous psychiatric diagnosis [16]. Additional participants in W3, (*n* = 4956) aged 20–30 years were invited and 764 individuals responded. Hence the overall cohort that was followed was *N* = 24413 (with a sub-cohort starting in 1998 (W1,*n* = 19457) and followed through W2 and W3, and a second cohort added in 2010 (W3,*n* = 4956) [17].

The study obtained ethics approval by the Swedish Ethical Review Authority (case-numbers: 96–260, 01-218, 03-302, 2009/880 − 31, 2012/808-3, 2021 − 00843, 2021 − 0247). After a complete description of the study to the subjects, written informed consent was obtained.

#### Depressive symptoms

The Major Depression Inventory (MDI) was used to assess depressive symptoms, which were based on responses in W1, W2 and W3. Validity of the MDI scale has been shown in clinical and non-clinical setting [18–20]. The Major Depression Inventory (MDI), which covers 10 core depressive symptoms operationalized through 12 items [18–20]. Participants reported how often they had experienced each symptom during the previous two weeks using a six-point scale: all the time (5), most of the time (4), slightly more than half of the time (3), slightly less than half of the time (2), some of the time (1), and never (0). A total sum score (range 0–50) was calculated, and the severity of depressive symptoms was categorized as: no depressive symptoms (MDI < 20), mild (20–24), moderate (25–29), and severe (≥ 30)³¹. In this study, severity of depressive symptoms was used as a specifier. The first incident of depression in the waves determined the severity of depressive symptoms i.e. mild, moderate or severe. If an answer was missing on items in the questionnaire the answer was treated conservatively as 0 and added to the points of the other items. In total 53 cases had missing values in all depression questions and were excluded from the analysis.

#### Anxious distress

Anxious distress was evaluated as a specifier of depressive symptoms based on DSM-5 criteria. Five symptoms were assessed: feeling keyed up or tense, feeling unusually restless, difficulty concentrating because of worry, fear that something awful may happen, and fear of losing control. These items correspond to the DSM-5 anxious distress specifier and were measured using 5- or 6-point Likert scales, where participants reported how often each symptom was experienced during the preceding 1–2 weeks. Cut-off values used to define the presence of each symptom are detailed in Appendix 1.

Severity categories were based on DSM-5: mild (two symptoms), moderate (three symptoms), and moderate-to-severe (four or five symptoms) [21]. Participants without depressive symptoms and without anxious distress served as the reference group for all analyses. The DSM-5 distinguishes severe anxious distress by requiring motor agitation in addition to four or five symptoms; however, because motor agitation was not captured in the PART questionnaires, we could not separately identify ‘severe’ anxious distress. Therefore, in accordance with previous population-based analyses, moderate and moderate-severe anxious distress categories were combined [21]. For completeness, we also identified a group of participants who met criteria for anxious distress symptoms but did not meet the MDI threshold for depressive symptoms. Although anxious distress is defined in DSM-5 as a specifier of depressive (or bipolar) episodes, this group was retained in the analysis to explore whether anxiety-related symptoms below the depressive symptom threshold were associated with ASCVD.

### ASCVD

All participants were followed in the National Patient Register (NPR) for the occurrence of ASCVD up until December 31, 2021. Linkage to health care registers was done using the unique personal identification number.

The presence of ASCVD at follow-up was assessed by discharge diagnoses from the National Patient Register (NPR) on hospitalization and visits to specialized outpatient hospital clinics according to ICD-10 codes. In this study, ASCVD was operationalized as a composite outcome including ischemic heart disease (I20–I25), stroke (I60–I67, I69), hypertensive heart disease (I11–I13), heart failure (I50), and peripheral vascular disease, embolism, and thrombosis (I73–I74), based on previous Swedish register-based studies using similar definitions [22, 23]. We acknowledge that some of these diagnoses (e.g. hypertensive heart disease and heart failure) are not exclusively atherosclerotic, but they are strongly associated with underlying atherosclerotic disease and are frequently used as part of composite ASCVD outcomes in population-based register research. For this reason, and to ensure comparability with previous studies, we retained this broader ASCVD definition while also presenting sub-analyses for ischemic heart disease and stroke separately [24]. In addition to the register information, self-reports at the different waves regarding current and previous treatment for or diagnosis by a physician of angina pectoris, myocardial infarction and stroke were included in the ASCVD outcome. Premature ASCVD was defined as an ASCVD diagnosis with an onset before the age of 55 years for men and 65 years for women [25].

#### Covariates

Several variables were considered as confounders: age, self-reported gender, medical history of hypertension and /or diabetes and socio-economic status at baseline since these covariates previously has shown to be associated with both depressive symptoms and ASCVDs [26–28].

In addition, smoking, hazardous alcohol use, BMI and sedentary lifestyle were considered as covariates [29–32]. These factors are known to increase the risk for depressive symptoms and atherosclerotic cardiovascular disease and may contribute to a causal pathway. Age at baseline was categorized into 20–30 years, 31–40 years, 41–50 years, 51–60 years and > 60 years.

Socio-economic status was grouped using occupational status according to the Nordic Standard Occupational Classification (NSOC) of 1989. Five groups were classified: high/intermediate level salaried employees; assistant non-manual employees; skilled workers; unskilled workers; and self-employed.

Sedentary lifestyle was defined as exercising less than three times per week based on self-reported physical activity.

Smoking status was based on self-reports and categorized into ; regular smokers, occasional smokers, previous smokers or never smokers [33]. Smoking status was only reported at W2, and W3.

Hazardous alcohol use was defined using the Alcohol Use Disorders Identification Test tool [34]. Answers were dichotomized based on ≥ 8 points representing hazardous alcohol use for men and ≥ 6 points for women [34, 35]. Baseline levels, i.e. responses in the first questionnaire were considered i.e. 1998 in W1 or 2010 for the cohort entering in W3, if information was missing data from the follow-up questionnaire was used if available.

Body mass index (BMI) was calculated based on the formula BMI = kg/m^2^, using self-reported height and weight on the 1998 sub-cohort in W1 and on the 2010 sub-cohort in W3.

If responses were missing in baseline wave, data from the follow-up questionnaire in W2 or W3 were used, as long as the available information was prior to the outcome. Otherwise, the data was coded as missing on that particular variable.

### Statistical analyses

Descriptive statistics were reported in frequency and percentage for categorical variables, and as mean and standard deviation (SD) for continues variables. Aside from depressive symptoms and anxious distress, missing values for co-variates were treated as a missing category in the analysis. The proportion of missing values for participants ranged from 0.3% for BMI to 15.9% socio-economic status. Chi-square test and t-test were used to compare men and women in regard to the covariates, depressive symptoms and anxious distress status.

Cox regression was used to estimate hazard ratios (HR) and 95% confidence intervals (95% CI). The analysis of the association between first incident depressive symptoms and ASVCD and premature ASCVD was adjusted for confounders. Sub-analyses were performed for type of ASCVD, i.e., ischemic heart disease and stroke separately, and severity of depressive symptoms and anxious distress as specifier. No depressive symptoms nor anxious distress were used as reference group. SPSS version 19.11 and SAS 9.3 were used for statistical analyses.

## Results

Table 1 shows baseline characteristics of the study participants (*n* = 11,175), of whom the majority were women (56.8%) and younger than 50 years (74.5%). A sedentary lifestyle (44.3%), hazardous alcohol use (27.0%), and regular smoking (12.6%) were common. Depressive symptoms were reported by 15.8% of participants, including 5.5% with severe symptoms. Compared to men, women had more depressive symptoms (19.8% vs. 10.5%) and anxiety distress, whereas men had more diabetes (2.4% vs. 1.5%) and hazardous alcohol use (28.4% vs. 26%).

**Table 1:** Baseline characteristics of study sample stratified by gender, *N* = 11,175

	Overall (*N* = 11175)	Women (*n* = 6345)	Men (*n* = 4830)	*P*-value^c^
Age group (in years), *n* (%)				
20–30	3691 (33.0)	2191 (34.5)	1500 (31.1)	< 0.001
31–40	2452 (21.9)	1365 (21.5)	1087 (22.5)	
41–50	2178 (19.5)	1233 (19.4)	945 (19.6)	
51–60	2247 (20.1)	1228 (19.4)	1019 (21.1)	
≥ 60	607 (5.4)	328 (5.2)	279 (5.8)	
Hypertension, *n* (%)	631 (5.6)	343 (5.4)	288 (6.0)	0.111
Diabetes, *n* (%)	214 (1.9)	96 (1.5)	118 (2.4)	< 0.001
Smoking, *n* (%)				
Regular	1403 (12.6)	891 (14.0)	512 (10.6)	< 0.001
Occasional	876 (7.8)	490 (7.7)	386 (8.0)	
Ex-smoker	2714 (24.3)	1617 (25.5)	1097 (22.7)	
Never	6182 (55.3)	3347 (52.8)	2835 (58.7)	
Hazardous alcohol use, *n* (%)	3021 (27.0)	1647 (26.0)	1374 (28.4)	< 0.01
Sedentary lifestyle, *n* (%)	4949 (44.3)	3028 (47.7)	1912 (39.8)	< 0.001
BMI, mean (SD) in kg/m^2^	24.1 (5.04)	23.6 (5.06)	24.9 (4.92)	< 0.001
Socio-economic status, occupation, *n* (%)				
Unskilled/semi skilled	1328 (11.9)	890 (14.0)	483 (9.1)	< 0.001
Skilled	643 (5.8)	265 (4.2)	378 (7.8)	
Assistant non-manual	1416 (12.7)	1025 (16.2)	391 (8.1)	
Self-employed	479 (4.3)	163 (2.6)	316 (6.5)	
High intermediate	3797 (34.0)	2106 (33.2)	1691 (35.0)	
Anxious distress^a^, *n* (%)				
None	9097 (81.4)	4921 (77.6)	4176 (86.5)	< 0.001
Mild	1230 (11.0)	828(13.0)	402 (8.3)	
Moderate to severe	848 (7.6)	596 (9.4)	252 (5.2)	
Depressive symptoms (MDI)^b^, *n* (%)				
No	9410 (84.2)	5087 (80.2)	4232 (89.5)	< 0.001
Yes	1765 (15.8)	1258 (19.8)	507 (10.5)	
Mild	710 (6.4)	515 (8.1)	195 (4.0)	
Moderate	438 (3.9)	305 (4.8)	133 (2.8)	
Severe	617 (5.5)	438 (6.9)	179 (3.7)	

^a^Anxious distress, showing none to one, two or three or more anxiety symptoms respectively: defined as the presence of at least two of the following symptoms during the previous two weeks: feeling keyed up or tense, feeling unusually restless, difficulty in concentrating because of worry, fear that something awful might happen and fear of losing self-control

^b^Depressive symptoms assessed by Major Depression Inventory

^c^Pearson chi square test

Table 2 presents hazard ratios (HRs) for ASCVD, IHD, and stroke by depressive symptom status. In the fully adjusted model (Model 3), participants with depressive symptoms had an increased risk of ASCVD (HR = 1.42, 95% CI 1.19–1.68), IHD (HR = 1.39, 95% CI 1.06–1.83), and stroke (HR = 1.53, 95% CI 1.15–2.05).

**Table 2:** Hazard ratios for atherosclerotic cardiovascular disease, ischemic heart disease and stroke by depressive symptoms status and severity of depressive symptoms and anxious distress

	All ASCVD (*n* = 1316), HR (95% CI)				Ischemic heart disease (*n* = 522), HR (95% CI)				Stroke (*n* = 444), HR (95% CI)			
	*n*	Model 1	Model 2	Model 3	*n*	Model 1	Model 2	Model 3	*n*	Model 1	Model 2	Model 3
Depressive symptoms												
No	1107	Ref.	Ref.	Ref.	438	Ref.	Ref.	Ref.	369	Ref.	Ref.	Ref.
Yes	209	1.41 (1.21–1.64)	1.38 (1.18–1.63)	1.42 (1.19–1.68)	84	1.43 (1.12–1.83)	1.47 (1.15–1.89)	1.39 (1.06–1.83)	75	1.46 (1.13–1.88)	1.38 (1.06–1.81)	1.53 (1.15–2.05)
Severity of depressive symptoms (MDI)												
No	1107	Ref.	Ref.	Ref.	438	Ref.	Ref.	Ref.	369	Ref.	Ref.	Ref.
Mild	84	1.39 (1.10–2.77)	1.45 (1.14–1.84)	1.42 (1.10–1.84)	39	1.69 (1.19–2.39)	1.74 (1.22–2.49)	1.62 (1.11–2.37)	26	1.25 (0.82–1.89)	1.41 (0.86–1.99)	1.36 (0.87–2.14)
Moderate	51	1.44 (1.08–1.90)	1.41 (1.05–1.89)	1.45 (1.07–1.96)	20	1.45 (0.92–2.28)	1.53 (0.97–2.40)	1.44 (0.89–2.34)	20	1.59 (1.01–2.51)	1.46 (0.90–2.37)	1.70 (1.04–2.78)
Severe	74	1.40 (1.11–1.78)	1.32 (1.03–1.69)	1.38 (1.06–1.81)	25	1.16 (0.77–1.76)	1.18 (0.78–1.79)	1.13 (0.71–1.79)	29	1.60 (1.09–2.34)	1.41 (0.94–2.11)	1.61 (1.04–2.49)
Depressive symptoms with/without anxious distress												
No depressive symptoms or anxious distress	1079	Ref.	Ref.	Ref.	420	Ref.	Ref.	Ref.	365	Ref.	Ref.	Ref.
No depressive symptoms with anxious distress	28	0.89 (0.61–1.30)	0.87 (0.60–1.27)	0.65 (0.40–1.06)	18	1.56 (0.97–2.50)	1.49 (0.93–2.39)	1.46 (0.85–2.51)	4	0.04 (0.13–0.94)	0.35 (0.13–0.93)	0.11 (0.02–0.80)
Depressive symptoms without anxious distress	62	1.43 (1.09–1.86)	1.38 (1.06–1.81)	1.36 (1.01–1.81)	29	1.86 (1.26–2.73)	1.81 (1.23–2.66)	1.88 (1.25–2.82)	22	1.40 (0.90–2.19)	1.36 (0.87–2.13)	1.40 (0.86–2.27)
Depressive symptoms with anxious distress	147	1.40 (1.17–1.67)	1.33 (1.11–1.59)	1.33 (1.12–1.65)	55	1.30 (0.97–1.74)	1.24 (0.93–1.67)	1.17 (0.84–1.62)	53	1.44 (1.07–1.93)	1.37 (1.02–1.85)	1.50 (1.09–2.07)

*ASCVD* Atherosclerotic cardiovascular disease, *HR* hazard ratio, *CI* confidence interval

Model 1 was adjusted for age and gender

Model 2 was further adjusted for hypertension, diabetes, and socio-economic status (occupation)

Model 3 was further adjusted for physical inactivity, hazardous alcohol use, smoking and BMI

When considering severity, mild (HR = 1.42, 95% CI 1.10–1.84), moderate (HR = 1.45, 95% CI 1.07–1.96) and severe (HR = 1.38, 95% CI 1.06–1.81) depressive symptoms were associated with increased ASCVD risk. Associations were also found for mild symptoms and IHD (HR = 1.62, 95% CI 1.11–2.37). Moderate (HR 1.70, 95% CI 1.04–2.78) and severe depressive symptoms (HR 1.61, 95% CI 1.04–2.49) were associated with stroke, whereas the association for mild depressive symptoms did not reach statistical significance.

When anxious distress was considered, both depressive symptoms without (HR = 1.36, 95% CI 1.01–1.81) and with anxious distress (HR = 1.33, 95% CI 1.12–1.55) were associated with higher ASCVD risk. Depressive symptoms without anxious distress were linked to IHD (HR = 1.88, 95% CI 1.25–2.82), while depressive symptoms with anxious distress were more strongly associated with stroke (HR = 1.50, 95% CI 1.09–2.07).

Table 3 shows results for premature ASCVD (*n* = 305). The association between depressive symptoms and premature ASCVD persisted after full adjustment (HR = 1.52, 95% CI 1.12–2.05). Among severity categories, the relationship remained significant only for mild depressive symptoms (HR = 1.61, 95% CI 1.05–2.48). Considering anxious distress, only depressive symptoms with anxious distress were associated with premature ASCVD (HR = 1.41, 95% CI 1.00–1.99).

**Table 3:** Hazard ratios for premature atherosclerotic cardiovascular disease by depressive symptoms and severity of depressive symptoms

	Premature ASCVD (*n* = 305), HR (95% CI)			
	*n*	Model 1	Model 2	Model 3
Depression				
No	231	Ref.	Ref.	Ref.
Yes	74	1.54 (1.17–2.01)	1.57 (1.18–2.07)	1.52 (1.12–2.05)
Severity of depressive symptoms (MDI)				
No	231	Ref.	Ref.	Ref.
Mild	31	1.59 (1.07–2.35)	1.70 (1.14–2.53)	1.61 (1.05–2.48)
Moderate	18	1.60 (0.99–2.59)	1.59 (0.97–2.62)	1.57 (0.94–2.63)
Severe	25	1.44 (0.95–2.20)	1.41 (0.91–2.17)	1.39 (0.88–2.20)
Depressive symptoms with and without anxious distress				
No depressive symptoms or anxious distress	221	Ref.	Ref.	Ref.
No depressive symptoms with anxious distress	10	1.05 (0.56–1.98)	1.00 (0.53–1.88)	0.62 (0.25–1.51)
Depressive symptoms without anxious distress	25	1.65 (1.07–2.53)	1.64 (1.07–2.53)	1.57 (0.98–2.51)
Depressive symptoms with anxious distress	49	1.49 (1.09–2.04)	1.40 (1.01–1.93)	1.41 (1.00–1.99)

Model 1 was adjusted for age and gender

Model 2 was further adjusted for hypertension, diabetes, and socio-economic status (occupation)

Model 3 was further adjusted for physical inactivity, hazardous alcohol use, smoking, and BMI

## Discussion

The findings from this longitudinal study show a consistent association between depressive symptoms and overall ASCVD and specific ASCVD (such as ischemic heart disease and stroke) emphasizing the independent contribution of depressive symptoms to cardiovascular outcomes. Exploration of severity of depressive symptoms indicated differential risk profiles for specific ASCVD subtypes: mild symptoms were more strongly associated with IHD, whereas moderate and severe symptoms showed greater linkage to stroke. Notably, the risk of premature ASCVD was particularly pronounced among individuals with mild depressive symptoms. Both depressed individuals with and without anxious distress demonstrated elevated ASCVD risk. Anxious distress, however, did not confer an additional risk for premature ASCVD beyond that of depressive symptoms alone.

In general, our findings align with global literature. A recent, comprehensive meta-analysis of 26 studies covering 1.9 million individuals reported a 1.28 times higher risk of myocardial infarction in patients with depressive symptoms with risk of all-cause mortality escalating to 43% [36]. Furthermore, another meta-analysis of 28 studies confirmed depressive symptoms to be an independent risk factor for the development of various forms of ASCVD, particularly myocardial infarction, with the odds of 1.60 [28]. More importantly, depressive symptoms has not only been associated with ASCVD but also reported to result in adverse clinical outcomes, with one meta-analysis reported that patients with depressive symptoms faced a 1.89-fold greater risk of major adverse cardiac events following percutaneous coronary interventions [37].

Our findings are broadly in line with previous studies, including earlier analyses from the PART cohort and large international meta-analyses, which have shown that depressive symptoms are associated with increased cardiovascular risk [12, 13, 36, 38]. Our current study, with investigation into premature ASCVD, adds an important new dimension, recognizing the role of depressive symptoms in early onset ASCVD in the Swedish population. Our findings show that depressive symptoms increase risk of premature ASCVD in a similar effect size as for ASCVD occurring later in life (non-premature ASCVD). A study in the US, like ours, has shown an association between poor mental health and cardiovascular disease among young adults, i.e. 49 years and younger. Psychosocial stressors and dysregulation of neurohormonal pathways have been suggested as potential mechanisms linking depressive symptoms to an increased risk of cardiovascular diseases in younger adults [39].

There is a consensus that severe forms of depressive symptoms should be a considered a tier II moderate-risk condition that predisposes to accelerated atherosclerosis and premature ASCVD [40]. A systematic review of 101 studies also suggested that clinical features of depressive symptoms, such as the intensity, recurrence, and duration of depressive episodes, might influence the link between depressive symptoms and cardiovascular disease [41]. Another large study tracing participants over a 13-year survey established that mild and major depressive symptoms were associated with an elevated 10-year ASCVD risk and significantly higher lifetime ASCVD risk in younger populations [42]. Our study is in agreement with the above reports. Although all levels of depressive symptom severity were associated with increased ASCVD risk, we did not observe a clear dose–response pattern across severity categories. The adjusted hazard ratios from our study suggest that even mild depressive symptoms are associated with a heightened risk of ischemic heart disease which supports the hypothesis that even subclinical levels of depressive symptoms are associated with specific cardiovascular conditions. This finding aligns with above mentioned studies that highlighted depressive symptoms as a potential contributor to cardiovascular risk, independent of traditional risk factors. However, our results extend this by demonstrating that the risk varies by the type of ASCVD and concomitant anxious distress.

The presence of anxious distress appeared to modify the risk associated with depressive symptoms. Although the confidence intervals overlap, suggesting no statistically significant difference between the associations, participants with both depressive symptoms and anxious distress showed an 50% increased risk of stroke, while participants with depressive symptoms but without anxious distress exhibited a higher likelihood of ischemic heart disease. These findings resonate with a large UK Biobank study of 431,973 participants which also reported higher hazard ratios for stroke as compared to ischemic heart disease in patients with concurrent depressive symptoms and anxiety [43]. Further, a meta-analysis encompassing 48 studies also reported a higher pooled prevalence of anxiety (13.5% vs. 9.1%) in stroke patients as compared to ischemic heart disease [44]. The lack of statistical significance for some subgroup associations, particularly for premature ASCVD and smaller severity or anxious distress categories, likely reflects limited statistical power. Yet, the effects presented, estimates, and direction of these associations are in line with the results from our other analyses. In addition, we examined a small group with anxious distress symptoms but without MDI-defined depressive symptoms. As anxious distress is conceptually a specifier of depressive episodes, findings in this group should be interpreted cautiously and were not the primary focus of the study. Further studies are needed to the association between populations with depression and anxiety and premature ASCVD.

### Strengths and limitations

This study contributes to the growing body of research exploring the link between depressive symptoms and premature ASCVD, adding to recent insights in this area. A key strength of this study is its longitudinal design, which integrates self-reported data with registry-based information. Register information is complete across the three waves for participants, and potential concerns regarding retention should thus be limited. Additionally, the use of the validated MDI enabled a reliable assessment of both the presence and severity of depressive symptoms [19].

Despite the contributions of this study, certain limitations must be acknowledged. The response rates at baseline and follow-up were low, particularly among individuals with psychiatric disorders, which may introduce selection bias and affect the external validity of the findings [45].

The small number of premature ASCVD cases do limit the power for some subgroup analyses, particularly groups involving moderate or severe depressive symptoms and the group with depressive symptoms without anxious distress. These associations need to be interpreted with caution.

Furthermore, our ASCVD definition was relatively broad and included diagnoses that are not purely atherosclerotic, such as hypertensive heart disease and heart failure, which may introduce some heterogeneity in the outcome; however, sub-analyses for ischemic heart disease and stroke provided more specific atherosclerotic endpoints.

Self-reported data for mental health and lifestyle factors like physical activity introduces the possibility of recall bias, which may lead to both over- and underestimation. For symptoms of mental health, participants may have either overreported symptoms due to heightened self-awareness or distress at the time of filling in the questionnaire or underreported due to stigma, memory limitations, or a lack of recognition of their symptoms [46]. In regards to lifestyle factors, individuals may tend to underreport hazardous behaviors due to potential stigmatization as well [47]. This variability in recall could influence the observed associations between depressive symptoms and cardiovascular outcomes. The treatment and diagnoses of diabetes and hypertension were also assessed based on self-reported information. Given their initially asymptomatic nature, these conditions may have been underestimated, potentially influencing the observed associations. Furthermore, the study did not assess changes in depressive symptoms over time, meaning it was unable to capture the potential progression or remission of symptoms. Some individuals may have experienced worsening depressive symptoms after the first measured incident in the following waves, while others may have recovered across time due to treatment or natural recovery, and others may later even have relapsed. The absence of longitudinal symptom tracking limits our ability to determine whether persistent or fluctuating depressive symptoms contribute differently to cardiovascular risk. As a result, further studies employing time-sensitive analyses are warranted to clarify the temporal relationship between depressive symptoms and premature ASCVD. Finally, as an observational study, causal inferences cannot be drawn, underscoring the need for further research to confirm these findings.

## Conclusion

In this study, using a population-based cohort with up to 23 years of follow up, we found an association between depressive symptoms, and the risk of ASCVD and premature ASCVD irrespectively of the severity of the depressive symptoms or comorbidity with anxious distress. The comprehensive exploration of anxiety, depressive symptoms, and their interplay with cardiovascular outcomes underscores the need for holistic approaches to mental and cardiovascular health. In addition, it underlines the need of addressing depressive symptoms that may contribute to premature ASCVD.

## Supplementary Information

Supplementary Material 1.

## Data Availability

Data are ethically restricted for patient privacy concerns. However, de-identified, participant level data can be obtained pending ethical approval.

## Abbreviations

ASCVD: Atherosclerotic cardiovascular disease
BMI: Body mass index
DALYs: disability-adjusted life years
MDI: Major depression inventory
NPR: National Patient Register
PART: PART study (In Swedish short for: Psykisk hälsa, Arbete och RelaTioner)
SD: Standard Deviation
