# Supplementary material for: Association between depressive symptoms and atherosclerotic cardiovascular disease: a Swedish population-based cohort study
Source: BMC Cardiovasc Disord. 2026 May 13;26:417. doi: 10.1186/s12872-026-05629-8 (PMC13173858; doi:10.1186/s12872-026-05629-8)
Supplement: Supplementary file 1 — Supplementary Material 1. [file 12872_2026_5629_MOESM1_ESM.doc]

**Appendix 1. DSM-5 Criteria for Anxious Distress, Mental Health Scales in the PART Study, and Assessment Questions**

Adapted from Aysha Almas et al.

| DSM V criteria for anxious distress ^1^ | Scale | Question on Symptom (translated) |
| --- | --- | --- |
| Feeling keyed up or tense. | Psychological well-being scale^2^ | How many times have you felt calm and relaxed during the past week?  Those who replied “never” or “sometimes” were regarded as having anxious distress symptom |
| Feeling unusually restless. | Major depression inventory ^3^ | How many times have you felt very restless in past 2 weeks?  Those who answered “all the time”, “most of the time” or “slightly more than half of the time” were regarded as having anxious distress symptom |
| Difficulty concentrating because of worry. | Major depression inventory | How many times have you had difficulty in concentrating in past 2 weeks?  Those who answered “all the time”, “most of the time” or “slightly more than half of the time” were regarded as having anxious distress symptom |
| Fear that something awful may happen. | Symptoms of anxiety during the past 30 days.^4^ | How much in the past 1 month have you experienced fear of dying?  Those who answered “much” or  “very much” were regarded as having anxious distress symptom |
| Feeling that the individual might lose control of himself or herself. | Symptoms of anxiety during the past 30 days | How much in the past 1 month have you had fear of losing control?  Those who answered “much” or “very much” were regarded as having anxious distress symptom |

^1^ Anxious distress is defined as the presence of at least two of the following symptoms during the majority of days of a major depressive episode or persistent depressive disorder (dysthymia). In the present study severity was classified as; mild: two symptoms, moderate: three symptoms, moderate-severe: four or five symptoms; and severe: four or five symptoms and with motor agitation. (http://dsm.psychiatryonline.org//, doi: 10th April 2015)

^2^ Bech, P., Gudex, C., Staehr Johansen, K. (1996). The WHO (Ten) Well-Being Index: Validation in Diabetes. Psychother Psychosom, 65, 183-190.

^3^ Bech, P., Wermuth, L. (1998). Applicability and validity of the Major Depression Inventory in patients with Parkinson´s disease. Nord J Psychiatry, 52, 305-309

.^4^ Sheehan, D. V. (1983). The anxiety disease (pp 124-129). New York: Charles Scribners Sons
